# Potential protective effect of Tualang honey on BPA-induced ovarian toxicity in prepubertal rat

**DOI:** 10.1186/1472-6882-14-509

**Published:** 2014-12-17

**Authors:** Siti Sarah Mohamad Zaid, Shatrah Othman, Normadiah M Kassim

**Affiliations:** Department of Anatomy, Faculty of Medicine, University of Malaya, 50603 Kuala Lumpur, Malaysia; Department of Molecular Medicine, Faculty of Medicine, University of Malaya, 50603 Kuala Lumpur, Malaysia; Department of Environmental Sciences, Faculty of Environmental Studies, UPM Serdang, 43400 Selangor, Malaysia

**Keywords:** Tualang honey, Bisphenol-A, Ovary, Toxicity, Antioxidant

## Abstract

**Background:**

To investigate the potential protective effects of Tualang honey against the toxicity effects induced by Bisphenol A (BPA) on pubertal development of ovaries.

**Methods:**

This study was conducted on pre-pubertal female Sprague Dawley rats. Animals were divided into four groups (n = 8 in each group). Group I was administered with vehicle 0.2 ml of corn oil (Sigma-Aldrich, USA) using oral gavage daily for six weeks; these animals served as negative control (CO group), Group II was administered with BPA suspended in corn oil at 10 mg/kg body weight and served as positive control (PC group), Group III was administered with 200 mg/kg body weight of Tualang honey 30 min before the administration of BPA at 10 mg/kg (TH group) while Group IV was administered with 200 mg/kg body weight of Tualang honey 30 min before the administration of corn oil (THC group). Body weight of all animals were monitored weekly.

**Results:**

The BPA-exposed animals exhibited disruption of their estrus cycle, while those animals treated with BPA together with Tualang honey, exhibited an improvement in percentage of normal estrous cycle. Their ovaries had lower numbers of atretic follicles compared to the PC group but higher than the CO group.

**Conclusions:**

Tualang honey has a potential role in reducing BPA-induced ovarian toxicity by reducing the morphological abnormalities of the ovarian follicles and improving the normal estrous cycle.

## Background

In recent years, environmental toxicants have become a serious health concern. It has caused a rise in concern when exposure of endocrine-disrupting chemicals (EDCs) on human and wildlife has effects on the reproduction development and function [1, 2]. One of the EDCs is bisphenol A that is widely used in industries as plasticizer for the production of polycarbonate plastics and epoxy resins [3]. In daily life, bisphenol A is widely used in numerous products including digital media (CDs and DVDs), electronic equipment, automobiles, construction glazing, sports safety equipment, medical devices, tableware, reusable bottles, toys, water pipes and food containers [4, 5]. The dramatic increase of bisphenol A exposure in humans were detected in serum, follicular fluid and amniotic fluid [6], fetal serum [7], milk of nursing mother [8] and urine [9, 10]. These findings have resulted in both scientific and public interests in assessing bisphenol A as a potential EDCs to health risk.

Several studies have reported that bisphenol A could induce morphological and functional alterations of the female genital system, especially in the ovaries at low presumably environmentally relevant doses [11]. In vitro studies claimed that bisphenol A negatively affects granulosa cell steroidogenesis by altering the steroidogenic enzymes and stimulatory effects on vascular endothelial growth factor (VEGF) that cause uncontrolled neovascularization [5, 12]. Subsequently, these findings suggest that these cells are highly sensitive to bisphenol A. Other in vivo studies also indicated that neonatal exposure to bisphenol A reduced the pool of primordial follicles in rats [13] while in mice, prenatal exposure to bisphenol A caused increased antral follicles but reduced corpora lutea percentages [14]. Moreover, development of polycystic ovaries (PCOS) [15] and decreased luteinizing hormone (LH) were observed [16]. Bisphenol A exposure has been claimed to promote oxidative stress (OS) and inflammation in women [17]. Treatment with bisphenol A induced OS in various tissues of rodent [18] by decreasing antioxidant enzymes and increasing hydrogen peroxide and lipid peroxidation [19]. Several compounds with antioxidant properties have been studied extensively as a method to counter disease-associated OS [20]. With these concerns in mind, many studies have been focusing on the possible therapeutic and preventive measures to counter the effects of deleterious effects of bisphenol A on the reproductive system.

Tualang honey, a wild Malaysian honey, contains high antioxidant properties [21–24]. It has been shown to ameliorate OS in renal and pancreas of streptozotocin-induced diabetic rat [25, 26]. It was also reported to prevent uterine and vaginal atrophy [27] as well as prevent osteoporosis of bones [28], protects rat testis against damage and OS induced by cigarette smoke [29]. It was also reported to have antiproliferative effects on oral squamous cell carcinomas (OSCC), human osteosarcoma (HOS) [30] and keloid fibroblasts [31]. The beneficial effects in those positive findings were claimed to be due to the antioxidant properties of Tualang honey.

The aim of the present study was to investigate the effects of bisphenol A administered during prepubertal period on ovarian follicular development, estrous cyclicity and hormonal profile. Consequently, the protective effects of Tualang honey against the deleterious effects of bisphenol A toxicity on ovarian follicular development, estrous cyclicity and hormonal profile were investigated.

## Methods

### Tualang honey (Agromas, Malaysia)

Tualang honey, a wild multifloral honey was supplied by Federal Agricultural Marketing Authority (FAMA), under Ministry of Agriculture and Agro-Based Industry, Malaysia. It was collected from Apis dorsata’s beehive built on a giant tree (tall and big), *Koompassia excels* (locally known as Tualang tree) that grows in the rain forest of Kedah, Malaysia. The honey was filtered to remove solid particles, concentrated in an oven at 40°C and subjected to γ irradiation at 25 kGy at Sterilgamma (M) Sdn. Bhd. (Selangor, Malaysia). The water concentration of the honey was standardized by FAMA at 18%.

### Animal and experimental design

Thirty-two prepubertal female Sprague Dawley rats aged 21 (P21) days were obtained from the Animal Husbandry, Faculty of Medicine, University of Malaya. The experimental design and procedures were conducted under protocols in compliance with EU Directive 2010/63/EU that approved by the Animal Care and Committee (ACUC) of University of Malaya. The animals were maintained under the standard laboratory conditions (temperature 25 ± 2°C, 50 ± 15% relative humidity and normal photoperiod of 12 h dark and 12 h light) with free access to rat commercial pellet diet (Gold Coin Feedmills Pte. Ltd, Malaysia) and water ad libitum. To minimize additional exposures to EDCs, water was supplied in glass bottles with rubber stoppers surrounded by a steel ring. They were acclimatized to the laboratory environment for a week prior to the commencement of the experiments. At P28, the animals were weighed and randomly divided into four groups (n = 8 in each group). Group I was administered with vehicle 0.2 ml of corn oil (Sigma-Aldrich, USA) and served as negative control (CO group). Group II was administered with BPA (Sigma Aldrich, USA) suspended in corn oil at 10 mg/kg body weight and served as positive control (PC group). Group III was administered with 200 mg/kg body weight of Tualang honey 30 min before the administration of BPA at 10 mg/kg (TH group). Group IV was administered with 200 mg/kg body weight of Tualang honey 30 min before the administration of corn oil (THC group). The treatment was performed in the morning (between 09:00 and 10:00 AM) once daily by oral gavage (to mimic the most likely route of human exposure) for six consecutive weeks.

Tualang honey was freshly prepared every morning (to avoid oxidation of the antioxidants) by dissolving in deionized water. Justification for dose selection of BPA was based on previous studies (influenced morphological and biochemical parameters in reproductive system) [32–35]. The dose of Tualang honey used was based on previous study which showed positive biological effects on female reproductive organs and the dose used was equivalent to routine/normal dose (one table spoon) in adult human [27]. Throughout the administration period, daily body weight was recorded while vaginal smear was taken to determine the estrous phase. After the last treatment, the animals were sacrificed. Venous blood samples were collected by direct heart puncture under deeply ketamine anaesthesia (Troy Laboratories, Australia). Serum from blood samples were store at −80°C until analysis. The ovaries were weighed and immediately fixed in 10% buffered formalin for histopathological analysis.

### Histopathological analysis

The harvested ovaries were fixed by immersion in 10% buffered formalin for 24 hours. Subsequently, the ovaries were hydrated in a graded series of ethanol, clearance by xylene, embedded in paraffin and sectioned. Serial sections (5 μm thickness) at were obtained and mounted onto glass slide, deparaffinized in xylene, stained with hematoxylin and eosin (Sigma Aldrich, USA) and dehydrated in a graded series of ethanol, cleared in xylene and mounted with Canada Balsam (Sigma-Aldrich). Histopathological changes and morphometric analysis in each ovarian section was performed on 62 total fields areas that were measured with grid lines using NIS-Elements software. All sections were observed under a light microscope (Olympus CH-B145-2) attached to image analyzer (NIS-Elements Advanced Research, Nikon, Japan).

### Classification and quantification of ovarian follicles

Ovarian follicles were classified and counted according to the criteria described by Zhuang et al. [36] as follows:

i) Primary follicles: an oocyte surrounded by a single layer of cuboidal granulose cells in part or in entirety.
ii) Secondary follicles: surrounded by more than one layer of cuboidal granulose cells with no visible antrum.
iii) Antral follicles: identified by the presence of an antral space and cumulus granulose cell layer.
iv) Corpus luteum: formed only after ovulation and filled with lutein cells.
v) Atretic follicles: had abnormal structures such as inspissated follicular fluid, degenerated egg, disorganized and thickened granulosa layers or filled with organizing fibrinous material in the antrum.

### Assessment of estrous cycles

Estrous cycles of rats were determined by daily observation of vaginal smears (between 09:00 and 10:00 AM). Vaginal secretion was collected using a plastic pipette filled with approximately 0.2 ml of normal saline (NaCl 0.9%). The tip of the pipette was inserted into the vagina to a depth of 2–5 mm. Then, the normal saline was flushed into the vagina and returned into the pipette by gently squeezing and releasing the bulb of the pipette. These steps were repeated for three times before the sample was collected. Subsequently, a drop of the cell suspension was smeared onto a labelled glass slide. Unstained smear was observed under a light microscope, without the use of the condenser lens, with 10 x and 40 x objective lenses. The cytological appearance of vaginal smears determined the phase of the estrous cycle as follows:

The proestrous phase (twelve to fourteen hours) was defined by the predominance of nucleated epithelial cells; an estrous phase (twenty-five to twenty-seven hours) primarily consisted of anucleated cornified cells; a metestrous phase (six to eight hours) consisted of the same proportion among leucocytes, cornified and nucleated epithelial cells; and the diestrous phase (fifty-five to fifty-seven hours) primarily consisted of a predominance of leucocyctes.

The criteria implemented for determining cycle patterns:

1. Regular cycle (RC): denoted a 4 to 5-day estrous cycle in which the estrous phase was observed at least twice during the sampling period.
2. Persistent diestrous: or prolonged diestrous have four or more days of diestrous phase during most of the cycles.

### Assay of serum estradiol, FSH, LH and progesterone

After two hours at room temperature, clotted blood samples were centrifuged at 1,000 x g for 15 minutes, extracted serum were stored at −80°C until subsequent analysis. ELISA (Cusabio, USA) was used for measurement of the circulating levels of 17β-estradiol (E_2_), follicle stimulating hormones (FSH), luteinizing hormones (LH) and progesterone (P_4_). Each sample was run in duplicate. In brief, 50 μl each of the standards, control and serum samples were added to respective wells coated with estradiol (E_2_), follicle stimulating hormones (FSH), luteinizing hormones (LH) or progesterone (P_4_) antibody and incubated with 50 μl of enzyme conjugate for two hours at 37°C in oven (Echo Therm, USA). Subsequently, the wells were rinsed three times with distilled water and 50 μl of the substrate was added and incubated for 15 minutes at 37°C. Reactions were terminated using 50 μl of stop solution. The optical density (O.D) was measured at 450 nm using a microplate reader (BioTek, USA). For determination of each hormonal level, a standard curve was constructed by plotting a graph of the absorbance of each reference standard against its corresponding levels. The inter-assay and intra-assay variations were found to be less than 15%.

### Statistical analysis

All statistical evaluations were performed with Statistical Package for Social Sciences (SPSS Inc. Chicago, Illinois, USA version 18.0 for windows). Parametric variables (body weight, ovary weight, hormonal assay and follicular counting) were analyzed using one-way analysis of variance (ANOVA) followed by Bonferroni test for multiple comparisons to identify significant different between groups. Estrous cycle phase was analyzed with Fisher’s exact probability test. Values are reported as mean ± S.E.M. P < 0.05 was considered significant.

## Results

### Body weight and ovary weight

Analysis of body weight and the weight of selected organs in toxicological studies are sensitive indicators for adverse effects of treatments. Analysis of relative organ weight (normalized to absolute organ weight to body weight) is an important and accurate analytical endpoint for identification of harmful effects of chemicals on the organ weights [37]. The value of body weight gain, changes in body weight, ovary wet weight and ovary relative weight in experimental groups are shown in Table 1. The mean body weight gain in each group was obtained from the difference in values between the final body weight (at P71) and the initial body weight (at P28). This body weight gain was divided by the final body weight (at P71) to obtain the percentage to obtain the percentage of body weight change. The changes in body weight in PC group and TH group were slightly higher than CO group. However, the difference was not significant. It was also noted that treatment with Tualang honey in BPA-exposed rats cannot prevent increase of body weight. The changes in body weight in THC group were comparable to CO group. The ovary relative weight in PC group was significantly higher than CO group. Interestingly, treatment with Tualang honey in BPA-exposed rat (TH group) showed significant reduction in body weight compared to the PC group.

**Table 1:** **Body weight and ovary weight in experimental groups**

Group	Body weight gain (g)	% changes in body weight	Ovary wet weight (g)	Ovary relative weight (mg/g body weight)
CO	78.88 ± 14.61	48.15 ± 6.60	36.88 ± 1.88	0.24 ± 0.01^b^
PC	99.25 ± 9.90	56.52 ± 3.49	49.38 ± 1.13	0.29 ± 0.12^a^
TH	92.50 ± 4.62	56.12 ± 1.94	40.00 ± 1.64	0.24 ± 0.02^b^
THC	89.5 ± 10.64	52.39 ± 3.47	38.75 ± 0.82	0.23 ± 0.01^bb^

The data were presented as mean ± S.E.M. Ovary relative weight with different superscripts are significantly different. ^a^P < 0.05 versus negative control group (CO) and ^b^P < 0.05, ^bb^P < 0.01 versus positive control group (PC). There were no significant changes in body weight between all groups.

CO = Negative control group administered with vehicle (corn oil).

PC = Positive control group administered with BPA at 10 mg/kg body weight.

TH = Tualang honey group administered with Tualang honey at 200 mg/kg body weight + BPA at 10 mg/kg body weight.

THC = Tualang honey control group administered with Tualang honey at 200 mg/kg body weight.

### Estrous cycle

Table 2 shows the estrous cycle patterns in all experimental groups. In CO group and THC groups, all rats maintained in normal estrous cycle. In PC group, only 37.5% presented normal estrous cycles and a higher percentage showed persistent diestrous (62.5%). These estrous cycles pattern were significantly different with both CO group and THC group. However, treatment with Tualang honey in BPA-exposed rats showed higher percentage in normal estrous cycle (62.5%) and lower in persistent diestrous (37.5%) compared to PC group but not statistically different.

**Table 2:** **Pattern of estrous cycle of rats from day 41 to day-70 in all experimental groups**

Group	Number of rats with normal cycles (%)	Persistent diestrous (%)
CO	8 (100%)^b^	0^b^
PC	3 (37.5%)^a^	5 (62.5%)^a^
TH	5 (62.5%)	3 (37.5%)
THC	8 (100%)^b^	0^b^

The data were presented as number (percentage). Numbers with different superscripts are significantly different. ^a^P < 0.05 versus negative control group (CO) and ^b^P < 0.05 versus positive control group (PC).

CO = Negative control group administered with vehicle (corn oil).

PC = Positive control group administered with BPA at 10 mg/kg body weight.

TH = Tualang honey group administered with Tualang honey at 200 mg/kg body weight + BPA at 10 mg/kg body weight.

THC = Tualang honey control group administered with Tualang honey at 200 mg/kg body weight.

### Follicle stimulating hormone (FSH) and luteinizing hormone (LH)

**Figure 1:**
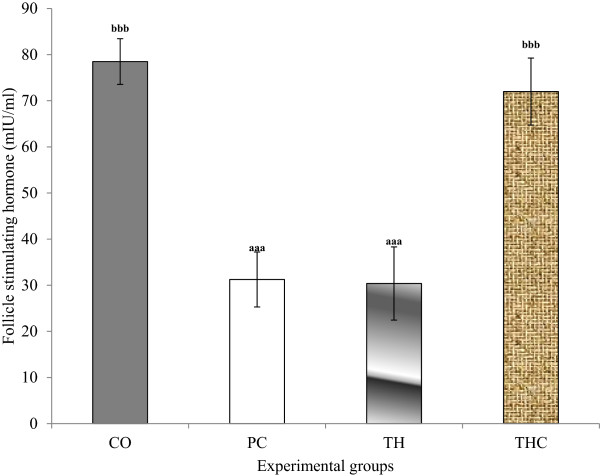
**Serum follicle stimulating hormone (FSH) in all experimental groups.** The data were presented as Mean ± S.E.M. Mean with different superscripts are significantly different. ^a^P < 0.001 versus negative control group (CO) and ^b^P < 0.001 versus positive control group (PC). CO = Negative control group administered with vehicle (corn oil). PC = Positive control group administered with BPA at 10 mg/kg body weight. TH = Tualang honey group administered with Tualang honey at 200 mg/kg body weight + BPA at 10 mg/kg body weight. THC = Tualang honey control group administered with Tualang honey at 200 mg/kg body weight.

**Figure 2:**
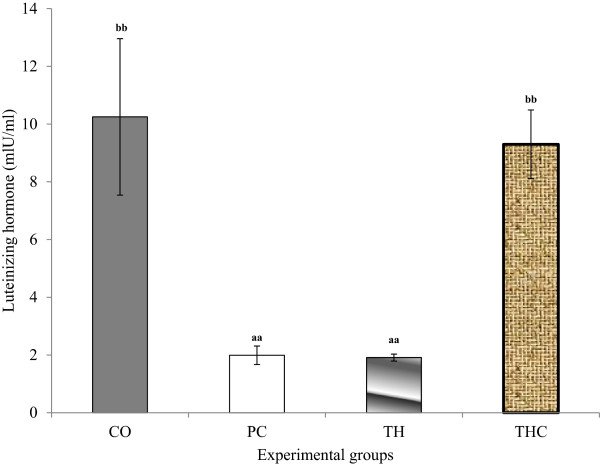
**Serum luteinizing hormone (LH) levels in all experimental groups.** The data were presented as Mean ± S.E.M. Mean with different superscripts are significantly different ^a^P < 0.01 versus negative control group (CO) and ^b^P < 0.01 versus positive control group (PC). CO = Negative control group administered with vehicle (corn oil). PC = Positive control group administered with BPA at 10 mg/kg body weight. TH = Tualang honey group administered with Tualang honey at 200 mg/kg body weight + BPA at 10 mg/kg body weight. THC = Tualang honey control group administered with Tualang honey at 200 mg/kg body weight.

Serum FSH and LH levels were significantly reduced in PC group and TH group compared to CO group and THC group. It was also noted that Tualang honey treatment on BPA-exposed rats cannot prevent the reduction of both hormones levels (Figures 1 and 2).

### 17β-Estradiol (E_2_) and progesterone (P_4_)

**Figure 3:**
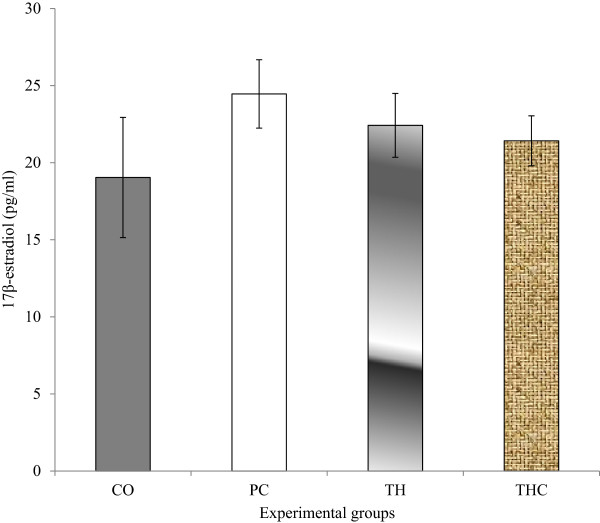
**Serum estradiol (E**_**2**_ **) levels in all experimental groups.** The data were presented as mean ± S.E.M. There were no significant differences in estradiol levels between all experimental groups. CO = Negative control group administered with vehicle (corn oil). PC = Positive control group administered with BPA at 10 mg/kg body weight. TH = Tualang honey group administered with Tualang honey at 200 mg/kg body weight + BPA at 10 mg/kg body weight. THC = Tualang honey control group administered with Tualang honey at 200 mg/kg body weight.

**Figure 4:**
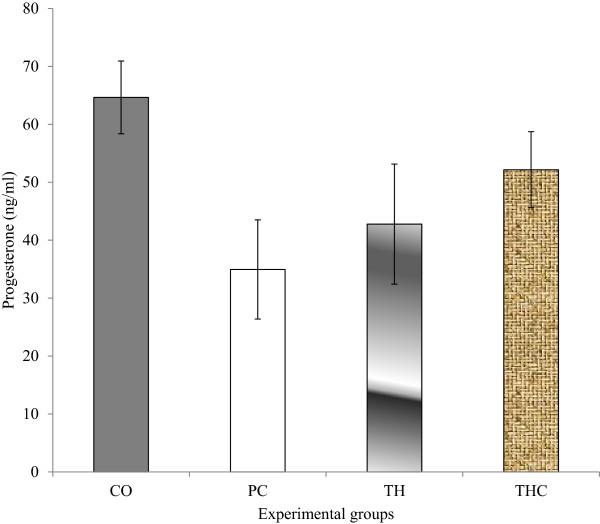
**Serum progesterone (P**_**4**_ **) levels in all experimental groups.** The data were presented as Mean ± S.E.M. There were no significant difference in progesterone levels between all experimental groups. CO = Negative control group administered with vehicle (corn oil). PC = Positive control group administered with BPA at 10 mg/kg body weight. TH = Tualang honey group administered with Tualang honey at 200 mg/kg body weight + BPA at 10 mg/kg body weight. THC = Tualang honey control group administered with Tualang honey at 200 mg/kg body weight.

There were no significant change with regards to both E_2_ and P_4_ levels in all experimental groups. However, the E_2_ level in PC group and TH group were slightly increased compared to the CO group (vehicle-treated). The reduction in the P_4_ level as seen in the PC group (BPA-treated) was slightly prevented with Tualang honey treatment (Figures 3 and 4).

### Ovarian follicular development

**Figure 5:**
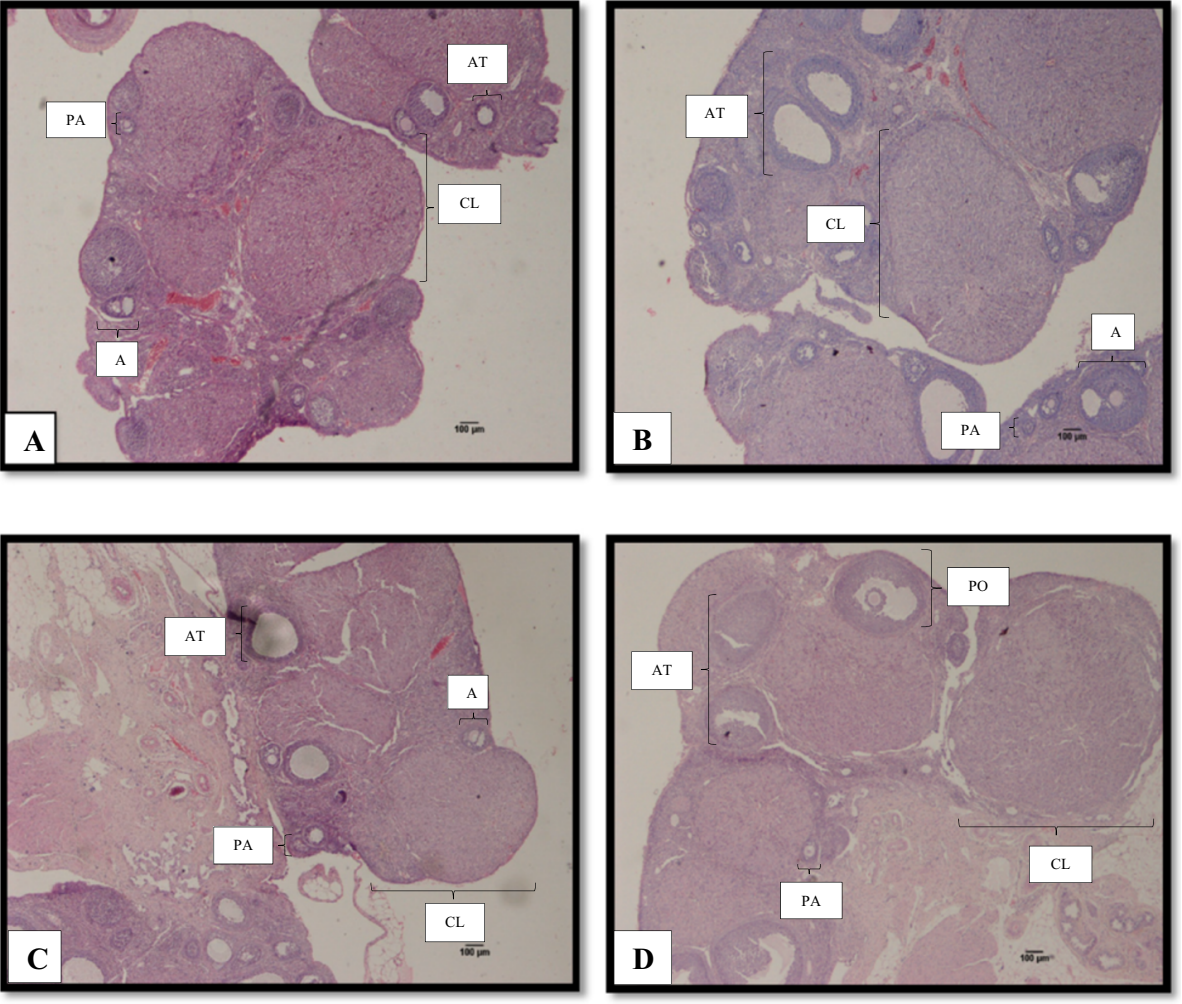
**Photomicrographs of representative ovarian section in experimental groups. A)** CO group **B)** PC group **C)** TH group **D)** THC group. Staining with H&E. PA: Preantral; A: Antral; At: Atretic; CL: Corpus luteum; PO: Preovulatory. Scale bar = 100 μm. CO = Negative control group administered with vehicle (corn oil). PC = Positive control group administered with BPA at 10 mg/kg body weight. TH = Tualang honey group administered with Tualang honey at 200 mg/kg body weight + BPA at 10 mg/kg body weight. THC = Tualang honey control group administered with Tualang honey at 200 mg/kg body weight.

**Figure 6:**
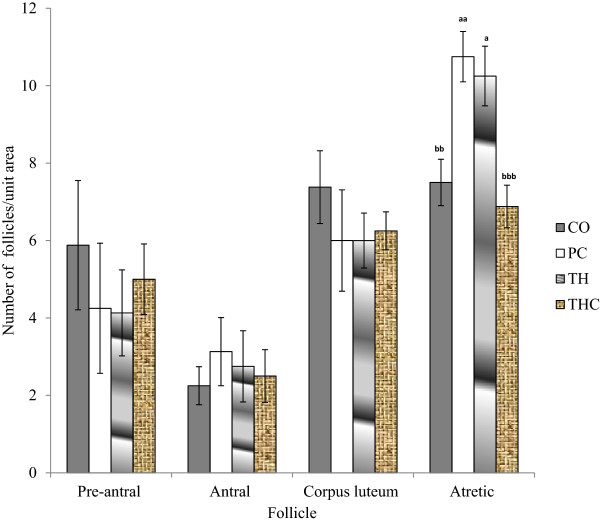
**Number of different types of follicles in all experimental groups.** The data were presented as Mean ± S.E.M. Means with different superscripts are significantly different. ^a^P < 0.05, ^aa^P < 0.01 versus negative control group (CO) and ^bb^P < 0.01, ^bbb^P < 0.001 versus positive control group (PC). There were no significant differences in pre-antral, antral and corpus luteum between all groups. CO = Negative control group administered with vehicle (corn oil). PC = Positive control group administered with BPA at 10 mg/kg body weight. TH = Tualang honey group administered with Tualang honey at 200 mg/kg body weight + BPA at 10 mg/kg body weight. THC = Tualang honey control group administered with Tualang honey at 200 mg/kg body weight.

Ovarian morphology analysis was conducted by qualitative and quantitative methods. Generally, ovaries from all groups displayed all stages of follicular development. Qualitative histological examination revealed that THC group exhibited healthy ovaries comparable to the ovaries of the negative control group (Figure 5). On the other hand, both the PC group and TH group showed some abnormalities of the ovaries with large antral-like follicles that did not arrive at ovulation and the presence of atretic cystic-like follicles. There was less number of corpus lutea observed. However, the degree of abnormalities was more apparent in the ovaries of the PC group compared to the TH group. Ovarian follicles at different stages were found in the ovaries of experimental groups. No significant difference was observed in the number of preantral, antral follicles and corpora lutea among all experimental groups (Figure 6). However, although not statistically different, the numbers of preantral follicles were lower in PC group and TH group compared to both control groups (CO group and THC group). The number of antral follicles were higher in PC group and TH group compared to both control groups. However, the numbers of antral follicles in TH group was slightly lower than PC group. Similar trend as in preantral follicles was also observed in the numbers of corpus luteum follicles. Only the number of atretic follicles in PC group and TH group were significantly higher than both control groups. However, the numbers of atretic follicles in TH group was slightly lower than PC group.

## Discussion

The prepubertal period is a crucial time during child development. According to the schedule of comparative age categories based on reproductive development in rat and human [38] the child period (post-weaning age) in rat begins at 21-day to 45-day of age which is 2-year to 12-year of age in human. The adolescence period in rat and human begin at 45-day to 90-day and 12-year to 16-year of age, respectively. The post-weaning age is a critical period of various neuroendocrine developments where the hypothalamus-pituitary-gonadal axis is still immature and therefore the levels of sex hormones in the body are relatively low [39]. This is why children are more susceptible to the toxic effects of BPA since bisphenol A toxicity may interfere with the maturation of hypothalamus-pituitary-gonadal axis through a variety of pathway including food and drinking water. Since BPA was detected in infant formula [40, 41] and leaching from polycarbonate baby bottles during washing, boiling and brushing [42, 43], the general population has become more concern over BPA exposure and its potential effects.

Several studies have reported that BPA exposure in rodent models correlates with weight gain [14, 16, 44] although other studies have not found this correlation [45–47]. In the present study, BPA caused a slight increase in the body weight though not significantly different from the CO rats. Thus, our results were in agreement with the latter studies. The discrepancy in these results could be due to differences in the sensitivity of the strain used to BPA exposure [48], dose selection, route of exposure, time window of exposure (age) and duration of exposure [49, 50].

The term ‘estrous’ refers to the special period of sexual desire of the female rats [51]. Generally, the sexual maturation (puberty) of female rats occurs between six to eight weeks of age (42 to 56-old days) and assessed by the formation of the external orifice of the vaginal canal [52] where the first estrous cycle begins approximately one week after vaginal opening. The onset of puberty results from establishment of both amplitude and frequency of hypothalamic and pituitary hormones pulses that stimulate the synthesis and cyclic secretion of ovarian steroid hormone [51]. As reported in several studies [11, 14, 16, 44, 50, 53, 54], estrous cyclicity was disrupted by BPA in rodent models. It was observed that the estrous cycles became persistent diestrus, persistent estrous or ultimately to acyclicity. BPA-exposed rodents were also reported to have earlier onset of puberty based on vaginal opening. Our study also showed persistent diestrus and earlier onset of vaginal opening in BPA-exposed rats. Interestingly, we observed an improvement in the percentage of normal estrous cycle in BPA-exposed rats treated with Tualang honey.

Disruption of normal estrous cycle is an indicator to alteration in the function of the hypothalamic-pituitary axis in BPA-exposed female rats [16] by interfering with the normal production of gonadotrophin releasing hormone (GnRH) and thereby decreasing the secretion of FSH and LH. Anatomical evidence reveals that neurons in sexually dimorphic regions are responsible for primary hypothalamic signal for gonadotropin synthesis and secretion and the drive for LH surge required for ovulation [55, 56]. Study by Petersen and Barraclough (1989), described that sexually dimorphic region in the brain, namely the rostral preoptic area, is crucial for normal estrous cyclicity and estrogen positive feedback [57]. This region contains estrogen receptors (ERs) and aromatase that are able to convert *in situ* testosterone to estrogen in females or males during critical periods of sexual differentiation [58]. Thus, exogenous estrogenic compounds like BPA can influence this region during development by binding to ERs. In BPA-exposed female rats, the stimulation on hypothalamus was increased (hypothalamic maturation and an accelerated GnRH pulse frequency) and inhibition on pituitary (reduced LH levels). These resulted in earlier vaginal opening and first estrous cycle, suggesting the effects of accelerated hypothalamic maturation that occurred in early life due to BPA. In this study, it was found that treatment with Tualang honey could hinder the disruption in normal estrous cycle via the reversal of FSH and LH hormones to their normal levels, which is reflected in the normalization of GnRH production in the brain. These results are also in line with the improvement of morphological findings in the ovarian follicles. All these improvements could be explained by the fact that honey contains bioactive molecules that exert protective effects via their estrogenic properties, namely the flavonols. Flavonols are phytochemicals and originate from a subfamily of flavonoids with many biological activities. Quercetin and kaempferol are the two main naturally-occuring flavonols which share structural similarities with 17β-estradiol, and therefore have potential estrogenic effects [59]. Thus, their beneficial effect in the improvement of the function of hypothalamic-pituitary axis is possibly due to their ability to bind to ERs in rostral preoptic region of brain, competing with other xenoestrogenic like BPA. This hypothesis of the protective effects of flavonols via their estrogenic mechanism is also supported by previous studies [60, 61]. Indeed, it is possible that both flavonols in Tualang honey are accountable as the modulators of xenoestrogenic effects of BPA in the female reproductive functions.

In this study, decreased levels of serum LH were observed in BPA-treated rats compared to control group. The decrease in serum LH level may result in the formation of cystic follicle (anovulation follicle) and consequently lack of corpora lutea, reflecting reduction in the progesterone levels [12]. Cystic follicle is formed from anovulatory follicle surrounded by thin layers of granulose cells with non-detectable theca cell layers [62]. Others claimed that large antral-like follicles do not support ovulation process in the ovary [11]. Our results is in agreement with the previous study that the total numbers of follicles in BPA-exposed rats have a positive correlation with the ovarian weight [63]. The number of preantral follicles in BPA-exposed rat was reduced or less compared to control rat, indicating that exposure to BPA interfered with the normal development of growing follicles in the ovary. This result is similar to previous study that used prepubertal female rat as an animal model [34]. Even with the disruption in normal ovulation process and in persistent diestrous phase, some follicles still progressed but without ovulation, followed by atresia, as reflected by the increase in the number of atretic follicles in BPA-exposed rats compared to the control rat. Indeed, our results showed a trend towards an increase in the number of antral follicle as well as a decrease in the number of corpora lutea in BPA-treated rats compared to control group. These results are in agreement with previous finding that also showed reduction in the number of ovulated oocytes [14]. As for the BPA-exposed rats treated with Tualang honey, the morphological abnormalities of the ovary were reduced and the number of atretic follicles was also slightly lower.

Lipid peroxidation and generation of free radicals are the two major contributors for the toxicity effects of BPA [35]. Studies have also reported that BPA was shown to induce OS in different tissues rodents [19] and could promote OS and inflammation in women [17]. In particular, OS plays an important role in the pathologies of female infertility problems that influence the entire reproductive life span by affecting oocyte maturation, ovarian steroidegenesis, ovulation, luteolysis and luteal maintenance in pregnancy [64]. The metabolite groups of BPA such as quinones (phenolic precursors), metal complexes (complexors), aromatic nitro compounds (reduced hydroxylamine and nitroso derivatives) and conjugated imines (iminium species) can incorporate with electron transfer (ET) to induce ROS [65]. Small quantities of oxidized metabolite can act as catalyst in a redox manner with generation of large quantities of ROS that result in OS. Thus, ET-ROS-OS framework provide reasonable and convenience evidence that BPA is a toxic agent [65]. OS is an important exacerbating factor for diverse pathological processes which can lead to deoxyribonucleic acid (DNA) damage, mutations, cellular injury, oncogenesis and aging process [66]. The genotoxicity effects of BPA have been widely tested in vitro and in vivo studies [67, 68]. The comet assays shows that BPA caused DNA fragmentation in blood lympocytes and structural chromosome aberrations in bone-marrow cells [69]. Other studies claimed that BPA induce lipid peroxidation, DNA fragmention in spermatozoa and up-regulate clusterin expression in atrophic prostate epithelial cells [70], DNA damage in germ cells [71] and MCF-7 cells [72]. Recently, Tualang honey was reported for its anticancer activity in breast cancer cell lines via the upregulation of double strand DNA repair enzymes that preserve cellular DNA integrity [73] or by promoting apoptotic cell death [74]. In ultraviolet (UV) B radiation study, treatment with Tualang honey to PAM212 cells can result in the reduction of the number of cyclobutane pyrimidine dimers and 8-oxo-dG-positive cells (biomarkers of DNA damage) due to improvement in DNA repair [75]. In agreement with these findings, studies on Buckwheat honey [76] and honey from arid regions [77] also demonstrated their protective effects on DNA damage. In general, the reducing power, DPPH radical scavenging activity, metal chelating activity and hydroxyl radical scavenging activities are all attributed to the antioxidants mechanisms in honey in preventing DNA damage.

Interestingly, Tualang honey has also been claimed as an ideal antioxidant with both aqueous and lipophilic properties that enable it to easily penetrate biological membranes and consequently act at different cellular levels of the cells [22]. The combination of endogenous antioxidants and the antioxidants from natural products plays an important role in the protective antioxidant mechanism in the body system against OS. When BPA-exposed rats are treated with Tualang honey, there was a marked improvement in the morphological abnormalities in ovarian follicules. In addition to the reduced number of atretic follicles, there was also improvement in the percentage of animals with normal estrous cycle compared to BPA-exposed rats without Tualang honey treatment. These results illustrated the ability of Tualang honey as a potential antigenotoxic mediator that is able to reduce ovarian toxicity, hence demonstrating its contribution to the protective mechanism against the genotoxic effects induced by BPA.

## Conclusions

In conclusion, Tualang honey has a potential role in reducing BPA-induced ovarian toxicity by reducing the morphological abnormalities of the ovarian follicles and improving the normal estrous cycle.

## Authors’ information

NMK (PhD) is a Professor in Deparment of Anatomy, Faculty of Medicine, University of Malaya. Her areas of expertise including developmental anatomy, cytology (histology, electron microscopy, immunocytochemistry) and toxicology (endorine disrupting chemicals).

SO (PhD) is a senior lecturer in Department of Molecular Medicine, Faculty of Medicine, University of Malaya. Her areas of expertise including cellular immunology and drug discovery.

SMZ (Masters) is a postgraduate student in Department of Anatomy, Faculty of Medicine, University of Malaya. Her research project focus on preventing or ameliorating effects of natural products on BPA-induced reproductive toxicity in rats.

## Abbreviations

EDCs: Endocrine-disrupting chemicals
BPA: Bisphenol-A
VEGF: Vascular endothelial growth factor
PCOS: Polycystic ovaries
LH: Luteinizing hormone
FSH: Follicle stimulating hormone
E_2_: 17β-Estradiol
P_4_: Progesterone
OS: Oxidative stress
DNA: Deoxyribonucleic acid
DPPH: 1,1-diphenyl-2-pic-rylhydrazyl
OSCC: Oral squamous cell carcinoma
HOS: Human osteosarcoma
CO: Negative control group
PC: Positive control group
TH: Tualang honey group
THC: Tualang honey control group.
